# Electroacupuncture Alleviates Inflammation of Dry Eye Diseases by Regulating the *α*7nAChR/NF-*κ*B Signaling Pathway

**DOI:** 10.1155/2021/6673610

**Published:** 2021-04-09

**Authors:** Ning Ding, Qingbo Wei, Weimin Deng, Xinyi Sun, Jie Zhang, Weiping Gao

**Affiliations:** ^1^Ophthalmology Department of Traditional Chinese Medicine, The First Clinical Medical College, Nanjing University of Traditional Chinese Medicine, Nanjing City, Jiangsu Province 210023, China; ^2^Affiliated Hospital of Nanjing University of Chinese Medicine, Nanjing City, Jiangsu Province 210029, China; ^3^Zhongda Hospital, Southeast University, Nanjing City, Jiangsu Province 210029, China; ^4^Yizheng Hospital of Chinese Medicine, Yizheng City, Jiangsu Province 211400, China

## Abstract

**Purpose:**

We tried to investigate whether electroacupuncture (EA) can reduce inflammation of dry eye disease (DED) by regulating *α*7nAChR and inhibiting the NF-*κ*B signaling pathway.

**Methods:**

Healthy New Zealand white rabbits were treated with scopolamine hydrobromide (Scop) for 21 consecutive days to establish the DED animal model. After 21 days, EA, fluorometholone (Flu), and *α*7nAChR antagonist (*α*-BGT) treatments were performed, and the Scop injection was continued until day 35. During treatment, the fluorescence staining of the corneal epithelium and the level of tear flow were observed. The influence of EA on the LG pathology and inflammatory factors ACh, *α*7nAChR, and NF-*κ*B was detected using the LG histopathology, transmission electron microscopy (TEM), cytokine protein chip technology, enzyme-linked immunosorbent assay (ELISA), and Western blot.

**Results:**

The EA stimulation can reduce the corneal epithelial damage and repair epithelial cell ultrastructure, promote the tear secretion, relieve the LG atrophy and decrease lipid droplet accumulation in LG acinar cell, and reduce the levels of inflammatory cytokines (i.e., IL-1, MIP-1b, TNF-*α*, and IL-8) in the LG. The protective effect of EA on the inflammation and the ocular surface is similar to the corticosteroid Flu. EA and Flu can upregulate the expression of the *α*7nAChR and downregulate the expression of NF-*κ*B. The *α*7nAChR antagonist *α*-BGT can reverse the protective effect of EA on the LG and the inhibitory effect on the NF-*κ*B pathway and the expression of inflammatory factors but cannot affect the expression of Flu on the NF-*κ*B pathway and inflammatory factors.

**Conclusion:**

These results prove that EA can alleviate DEDs by stimulating the acupoints around the eyes. These beneficial effects are related to the upregulation of *α*7nAChR and the downregulation of NF-*κ*B in the LG. The protective effect of LG is mediated through the anti-inflammatory pathway mediated by *α*7nAChR. EA can reduce the NF-*κ*B P65 nuclear transcription and reduce inflammatory factors by regulating *α*7nAChR. This expression indicates that the *α*7nAChR/NF-*κ*B signaling pathway may serve as a potential therapeutic target for EA to treat DEDs.

## 1. Introduction

The dry eye disease (DED), a common disease in ophthalmology, includes the tear film homeostasis, ocular surface inflammatory reaction, and damage as its main features and is accompanied by ocular discomfort symptoms of the multifactor ocular surface disease [1]. According to the survey, DED, which is a common disease worldwide, has a prevalence of 5%–50% [2] and affects the quality of human life.

Tears are secreted by lacrimal glands (LGs) to protect and support the ocular surface, and the lack of tears can cause an aqueous deficiency. The long-term chronic inflammatory activation of the LG leads to abnormal acinar and ductal cell death and functional impairment [3]. Studies have found that [4] in all LG inflammatory diseases, proinflammatory cytokines, such as IL-1*β* and TNF-*α*, commonly increase, continue to attack the LG by stimulating the recruitment and the proliferation of lymphocytes, and interfere with the normal function of the gland. This interference is also the main reason for the decrease in the tear secretion. The LG, as a highly developed innervated gland, is closely related to the parasympathetic nerve and the neurotransmitter acetylcholine (ACh). The cholinergic stimulates the secretion of the lacrimal protein and the tear fluid and the corneal sensory nerve to pass the parasympathetic reflex of the trigeminal nerve. The activated parasympathetic nerve stimulates the LG to secrete and release the neurotransmitter ACh. The ACh and the cholinergic signal receptor m3 muscarinic receptor (m3AChR) bind to control the secretion of protein, electrolytes, and water, and the use of muscarinic antagonists can prevent the tear secretion [5–8]. However, ACh can bind to mAChRs and nicotinic receptors (nAChRs) and plays a key role in the cholinergic anti-inflammatory pathway (CAP) [9].

Acupuncture, as a traditional Chinese medicine treatment, is widely used in the treatment of many diseases, such as renal interstitial fibrosis, allergic rhinitis, pain, cerebral ischemic injury and neuroinflammation, and vascular dementia [10–13]. These studies show that acupuncture has neuroprotective and anti-inflammatory effects. Interestingly, some experiments have found that the systemic anti-inflammatory effect of acupuncture is directly or indirectly mediated by the efferent vagus nerve activation and the macrophage inactivation. As such, acupuncture may activate the CAP and the vagus nerve to release ACh and bind to *α*7nAChRs on macrophages, thereby inhibiting the release of proinflammatory cytokines [14, 15]. However, many other signal transduction pathways are found in the anti-inflammatory effect of acupuncture in animal models [16–20].

Among the local treatment methods for dry eye diseases of aqueous deficiency, topical steroids (0.1% fluorometholone (Flu) and 0.5% loteprednoate), 0.05% CSA, and autologous serum eye drops are most commonly used clinically [21]. The effect of inflammation in DEDs makes topical corticosteroids a natural candidate for treatment, but the obvious side effects of the long-term use of corticosteroids (including secondary glaucoma, infection, and cataracts) limit their use. Many studies have found that although acupuncture is minimally invasive, its effectiveness and safety have been unanimously recognized. Acupuncture can promote the LG stimulation and the tear secretion and provide continuous relief from dry eyes [22–26]. Although research supports the use of acupuncture to treat DEDs, the mechanism by which acupuncture exerts its anti-inflammatory effect remains elusive, thereby limiting our understanding of acupuncture and further clinical treatment.

Electroacupuncture (EA) is a combination of acupuncture and electrophysiological effects and can increase the acupuncture sensation and reduce the workload of rotating the needle. EA is more regular than the conventional acupuncture technique and easy to repeat. Therefore, EA is now widely used in research and clinical settings. The EA waveform we have performed in this study is the dense wave. The excitatory effect is dominant during treatment and can increase metabolism, promote the blood circulation, improve the tissue nutrition, and eliminate the inflammatory edema.

Therefore, in this study, we have used a rabbit model treated with the muscarinic choline inhibitor scopolamine hydrobromide (Scop) to explore the protective effect of EA on the LG and its anti-inflammatory mechanism and provide a theoretical basis for the acupuncture treatment of DEDs.

## 2. Materials and Methods

### 2.1. Experimental Animals

Healthy New Zealand rabbits (male and female, 2–3 months old, weight = about 1.5 kg) were purchased from Qinglong Mountain Experimental Animal Farm (Nanjing, China) and raised in the pharmacology laboratory of Jiangsu Provincial Hospital of Traditional Chinese Medicine. The experimental animals were housed in ambient conditions (room temperature, 22°C ± 2°C; relative humidity, 60% ± 5%; and alternating 12-hour light-dark cycle). Water and standard feed were provided ad libitum. Prior to the experiment, the anterior segment of the eyes of all animals was examined and should have no abnormality, and the tear flow strip should be greater than 10 mm per 5 min. The experimental protocol was approved by the Animal Care and Use Committee of Nanjing University of Traditional Chinese Medicine (Approval ID: 201809A018). According to the Animal Experiment Guidelines of Nanjing University of Traditional Chinese Medicine, rabbits received humane care, and great efforts were made to reduce the number of animals.

### 2.2. Instruments and Reagents

The following instruments and reagents were used in this study: Scop (Chengdu Pufeide Biotech Co., LTD., JOT-10515, China); tear detection filter paper strip (Tianjin Jingming New Technology Development Co., LTD., China); corneal stain filter paper (Tianjin Jingming New Technology Development Co., LTD., China); Huatuo brand disposable sterile acupuncture needles (Suzhou Medical Device Factory, China); WQ1002 Han's electroacupuncture treatment device; Flu eye drops (0.1%, Shentian Pharma, J20180068, China); *α*-BGT (promoter, catalog no. pk-ca707-00010-1, Germany); RM2135 slicer (LEICA, Germany); DMLS2 optical microscope (LEICA, Germany); rabbit cytokine quantification array QAL-CYT-1 kit (RayBiotech, Inc., Norcross, GA, USA); BCA method (Pierce, no. 23227); ACh ELISA kit (Nanjing Jinyibai Biotechnology Co., Ltd., catalog no. JEB14612, China); *α*7nAChR ELISA kit (Nanjing Jinyibai Biotechnology Co., Ltd., catalog no. JEB14612, China), enzyme-labeled instrument (BioTek ELx800; BioTek Instruments, USA); transmission electron microscope (H-7000; Hitachi, Ltd., Tokyo, Japan), antibody phospho-NF-*κ*B p65 (1 : 1000; catalog no. 3033S; Cell Signaling Technology), NK-*κ*B p65 (1 : 1000; catalog no. 08101524A; ENZO), *β*-actin (1 : 1000; catalog no. sc-58679, Santa Cruz); HRP-conjugated goat anti-rabbit IgG (1 : 5000; FMS-RB01; FcMACS, CA); and gel imager (ChemiDoc XRS System; Bio-Rad Laboratories, Japan).

### 2.3. Experimental Procedures

The New Zealand white rabbits were randomly divided into eight groups, namely, the Con, Scop, sham acupuncture (Scop+Sham), EA (Scop+EA), Flu (Scop+Flu), *α*-BGT (Scop+*α*-BGT), Flu+*α*-BGT (Scop+Flu+*α*-BGT), and EA+*α*-BGT (Scop+EA+*α*-BGT) groups. Each group had six rabbits. The Con group received no treatment. The remaining seven groups of animals were injected subcutaneously with 2.0 mg/mL Scop four times a day (8:00, 11:00, 14:00, and 18:00) to induce dry eyes for 21 consecutive days and given different treatments. The Scop injection was maintained for 35 consecutive days until the end of the experiment. The Scop+EA group was given acupuncture treatment (Jingming BL1, Cuanzhu BL2, Sizhukong SJ23, Temple EX-HN5, Tongzilian GB1) on day 22, and the needle was retained for 15 minutes once a day for 14 consecutive days. EA adopted the density wave with frequency, pulse width, and intensity of 4 Hz/20 Hz, 0.5 ms, and 1 mA, respectively. The intensity was based on the slight twitching of the muscle at the acupuncture site. The acupoints in the Scop+Sham group were punctured using blunt needles as in the EA group without penetrating the acupuncture points once a day for 14 consecutive days. The Scop+Flu group was administered with Flu eye drops three times a day (8:00, 13:00, and 18:00) for 14 days after successful modeling. The specific *α*7nAChR antagonist *α*-BGT was injected into the rabbit ear vein at 4.0 *μ*g/kg daily for 14 consecutive days. The tear amount (Schirmer I test (SIt)) and the fluorescein staining score (FL) were measured on days 1, 7, 14, 21, 28, and 35, and experimental animals were euthanized on day 35.

### 2.4. Schirmer I Test (SIt)

The tear detection filter paper strip was folded at one end and placed into the conjunctival sac of the outer third of the rabbit's lower eyelid. After 5 min, the filter paper was collected, and the wetting length was measured from the folding point.

### 2.5. Corneal Fluorescein Staining Score (FL)

The corneal staining filter paper strip was placed into the lower eyelid fornix of the rabbit and wetted, and the fluorescein was rapidly and evenly distributed on the cornea though eye blink. The corneal epithelial injury was graded with the cobalt blue filter. The cornea was classified into four quadrants, and the score was determined and shown as follows: absent, 0; less than five spots, 1; more than five spots, 2; and large-area fluorescein plaque, 3. Finally, the score of each grade was added, and the full score was 12.

### 2.6. Optical Microscopy

After euthanasia, the LGs were collected and fixed in 4% paraformaldehyde for 24 hours. The LG size was 2 mm × 2 mm. Further dissection, paraffin embedding, RM2135 slicer, H&E staining, and DMLS2 optical microscopy were performed.

### 2.7. Transmission Electron Microscopy (TEM) Examination

Samples for TEM were fixed in 2.5% glutaraldehyde in 0.1 mol/L phosphate buffer (pH 7.4) and then postfixed in 1% osmium acid. Subsequent to dehydration with an ascending alcohol series, the samples were embedded in epoxy resin. Small sections (1 mm^3^) were cut from the middle area of the cornea. The sections were subjected to double staining with lead acetate and uranyl acetate and were observed using a transmission electron microscope.

### 2.8. Cytokine Quantification Array

Quantibody® Rabbit Cytokine Arrays were performed using the Qal-CYT-1 kit. The total protein was extracted from the LG by using the tissue protein extraction kit, and the protein concentration was determined using the BCA method. In accordance with the instructions of the manufacturer's kit, the expression levels of eight cytokines in eight groups of LG (including IL-1a, IL-1b, IL-8, IL-17A, IL-21, Leptin, MIP-1b, and TNF-*α*) were detected using Quantibody® Rabbit Cytokine Array and repeated thrice. The InnoScan 300 Microarray Scanner (Innopsys, France) was applied to scan signals by using the Cy3 excitation curves.

### 2.9. ELISA

The LG of rabbits was immediately collected after euthanasia. After rinsing with normal saline, the LG was fully homogenized in an ice bath, diluted with 300 *μ* saline, and centrifuged. The supernatant was collected and stored at −80°C for further experiments. ACh and *α*7nAChR were detected using the ELISA kit, and their contents were determined using a microplate reader.

### 2.10. Western Blot

Each group of LG protein was extracted using the RIPA lysate. The supernatant was centrifuged, and the total protein concentration in the supernatant was determined using the BCA method. The calculated loading amount was added to 10% SDS-PAGE gel for electrophoretic separation. After the electrophoresis, the gel was cut and transferred to the membrane according to the molecular weight of the protein. After the membrane was completed, 5% milk was blocked for 1 hour, and the membrane was incubated in phospho-NF-*κ*B p65, NK-*κ*B p65, *β*-actin, and actin at 4°C overnight; washed three times with 0.05% Tween-20 Tris buffer saline for 10 minutes each time next day; and incubated in the HRP-conjugated goat anti-rabbit IgG for 1 hour. The TBST was washed three times at 10 minutes each time. The membrane was covered with the ECL liquid and detected using the imager.

## 3. Statistical Methods

Data were expressed as mean ± SEM. Comparisons between means were carried out using one-way analysis of variance followed by Tukey's multiple comparison test. The statistical analysis was performed using the GraphPad Prism 8.0 (San Diego, CA, USA).

## 4. Results

### 4.1. EA Is Involved in the Protective Effect of the Corneal Epithelium

**Figure 1:**
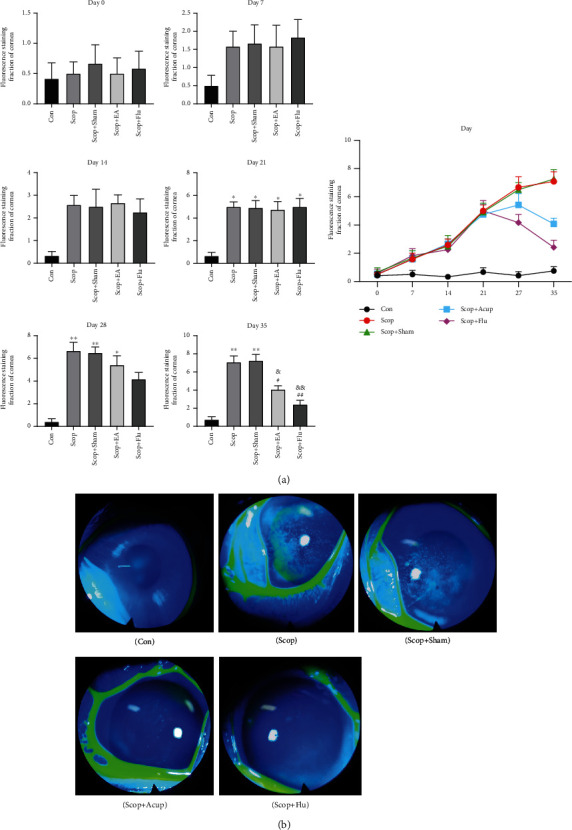
Effect of the electroacupuncture treatment on the corneal fluorescence staining in DED induced by scopolamine hydrobromide: (a) corneal fluorescence staining score; (b) corneal fluorescence staining on day 35. Quantitative data are expressed as mean ± SEM (*n* = 6). The Scop group has increased corneal fluorescence staining score significantly on day 21 when compared with the Con group. After 14 days of treatment, the EA and Scop+Flu groups have decreased corneal fluorescence staining score when compared with the Scop group, indicating that the corneal epithelial damage has been improved. ^∗^*P* < 0.05 and ^∗∗^*P* < 0.01 vs. the Con group; ^#^*P* < 0.05 and ^##^*P* < 0.01 vs. the Scop group; ^&&^*P* < 0.01 and ^&&^*P* < 0.01 vs. the Scop+Sham group.

Corneal fluorescence staining scores and corneal fluorescence staining imaging (*n* = 6) on day 35 were observed to detect whether EA was involved in the protective effect of the corneal epithelium. Compared with that of the Con group, the corneal fluorescence staining score of the Scop group increased significantly on day 21 (*P* < 0.05, Figure 1(a)). After seven days of treatment, the corneal fluorescence staining score of the EA group had no statistical difference with that of the Scop group (*P* > 0.05, Figure 1(a)). After 14 days of treatment, compared with that of the Scop group, the corneal fluorescence staining score of the EA group decreased (*P* < 0.05, Figure 1(a)), and the corneal staining score of the Scop+Flu group was significantly reduced (*P* < 0.01, Figure 1(a)), indicating that the corneal epithelial damage has been improved. The corneal fluorescent staining on day 35 is shown in Figure 1(b). The corneal epithelium of the rabbits in the Con group had almost no staining. The Scop and Scop+Sham groups were significantly stained. After treatment, the ocular surface staining of the EA and Scop+Flu groups decreased and scattered dotted dyeing. The above results showed that EA was involved in the protection of the corneal epithelium.

### 4.2. EA Treatment Leads to the Restoration of Corneal Epithelial Structure

**Figure 2:**
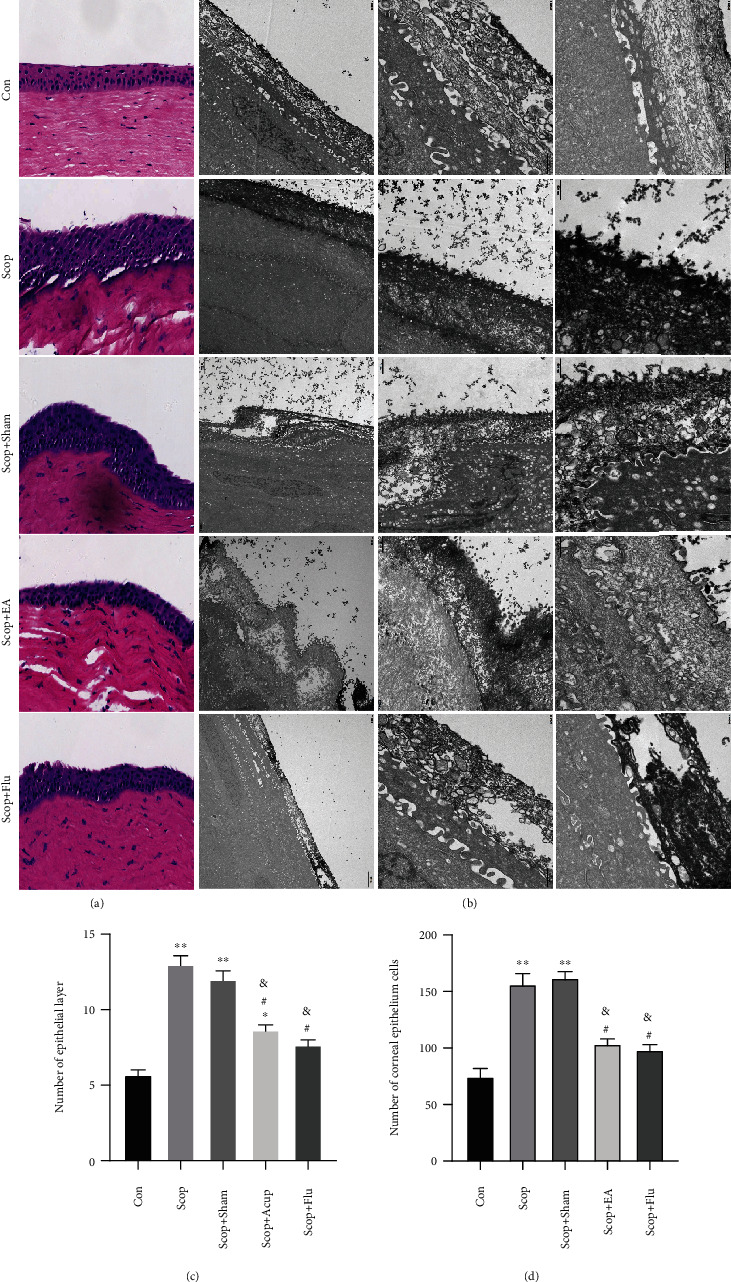
Alterations to the corneal epithelium following treatment. (a) Representative images demonstrating epithelial layers and epithelium cells in the cornea (hematoxylin-eosin staining, 20). (b) Corneal epithelial cells under transmission electron microscopy. (c) The number of epithelial layers. (d) The number of cells in the cornea epithelium was determined. ^∗^*P* < 0.05 and ^∗∗^*P* < 0.01 vs. the Con group; ^#^*P* < 0.05 vs. the Scop group; ^&^*P* < 0.05 vs. the Scop+Sham group.

The number of epithelial layers in the cornea was significantly increased in the Scop group compared with the number in the Con group (*P* < 0.01, Figures 2(a) and 2(c)). A significant increase in the number of corneal epithelium cells was also observed in the Scop group compared with the number in the Con group (*P* < 0.01, Figure 2(d)). Compared with that in the Scop group, the number of epithelial layers and corneal epithelium cells in the Scop+EA and Scop+Flu groups decreased significantly (*P* < 0.05, Figures 2(c) and 2(d)).

TEM evaluation of the corneal epithelial cells in the Con group revealed no abnormalities. By contrast, in the Scop group, the epithelial cells exhibited a loss of microvillus structures and epithelial cells, a widening of the intercellular space, severe expansion of rough endoplasmic reticulum, desmosome disintegration, and mitochondrial swelling (Figure 2(b)), this means a form of inflammation and even cell death. After EA stimulation, the epithelial cells exhibited sparse and short microvillus structures, mild rough endoplasmic reticulum expansion, and no obvious swelling of mitochondria, but the microvilli were still missing and missing locally (Figure 2(b)). The therapeutic effect of fluorometholone is similar to that of EA observed under the electron microscope.

### 4.3. EA Is Involved in the Protective Effect of the Lacrimal Glands

**Figure 3:**
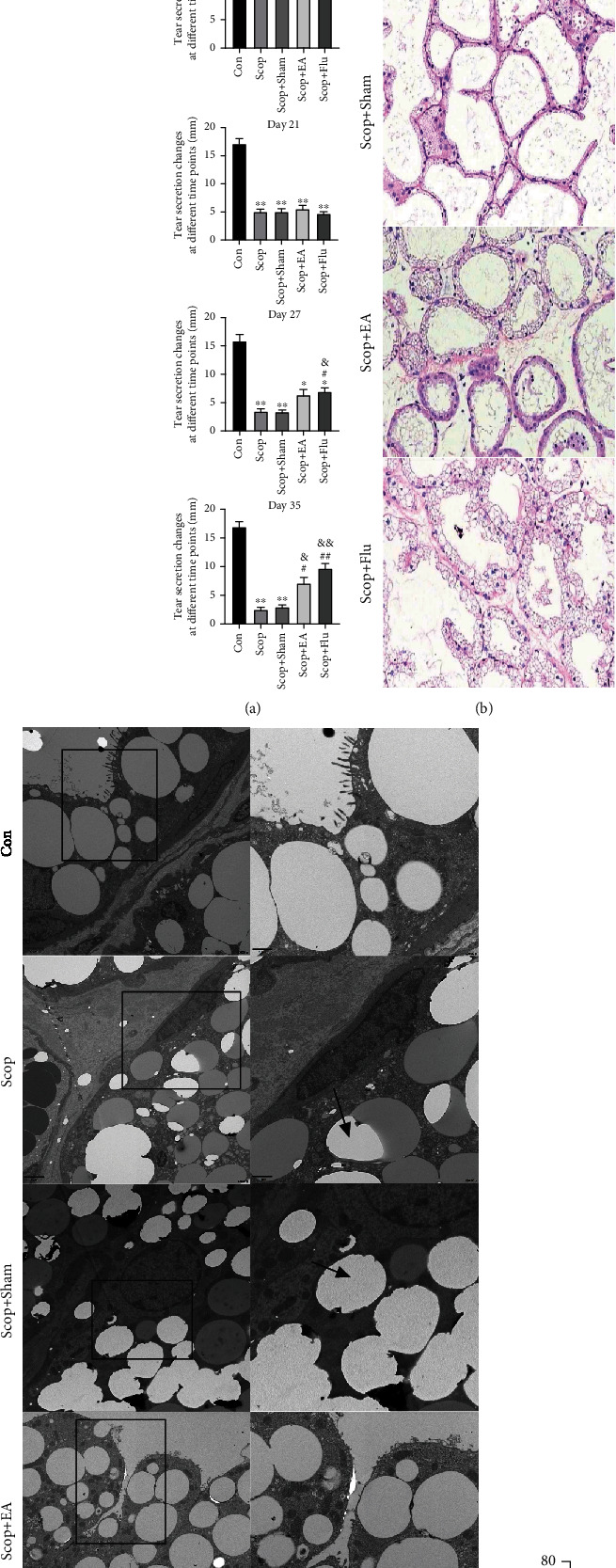
Effect of the electroacupuncture treatment on the tear flow and the lacrimal glands in the DED induced by scopolamine hydrobromide: (a) tear fluid flow (*n* = 6); (b) histopathological images of the cornea (hematoxylin-eosin staining, ×20) on day 35 (*n* = 3); (c) lacrimal glands under transmission electron microscopy; (d) the percentage of lacrimal gland area/total area (%). Quantitative data are expressed as mean ± SEM. ^∗^*P* < 0.05, ^∗∗^*P* < 0.01, and ^∗∗∗^*P* < 0.001 vs. the Con group; ^#^*P* < 0.05 and ^##^*P* < 0.01 vs. the Scop group; ^&^*P* < 0.05 and ^&&^*P* < 0.01 vs. the Scop+Sham group. (c) Ultrastructural analysis under transmission electron microscopy revealed that EA decreased lipid droplet accumulation in LG acinar cell. Lipid droplets (black arrows).

The tear flow measurement (*n* = 6) and the H&E staining on day 35 were performed to evaluate the area of the LG atrophy (*n* = 3) and detect the involvement of *α*7nAChR on the protective effect of EA on the LG. Compared with that in the Con group, the tear flow in the Scop group decreased markedly on day 21 (*P* < 0.01, Figure 3(a)). After seven days of treatment, compared with that in the Scop group, the tear flow in the EA group was not statistically different (*P* > 0.05), whereas compared with that in the Scop group, the tear flow in the Scop+Flu group increased (*P* < 0.05, Figure 3(a)). After 14 days of treatment, compared with that in the Scop group, the tear flow in the EA group increased (*P* < 0.05, Figure 3(a)), which indicated that the function of the LG improved.

The H&E staining on day 35 was performed to evaluate the area of the LG atrophy (*n* = 3): compared with that in the Con group, the LG atrophy area in the Scop group increased significantly (*P* < 0.001, Figures 3(b) and 3(d)). Compared with the Scop group, the EA group had decreased LG atrophy area after the EA stimulation (*P* < 0.05, Figures 3(b) and 3(d)), and the Scop+Flu group had decreased lymphatic infiltration of the LG.

Ultrastructural analysis using TEM revealed the presence of lipid droplet accumulation, which was located close to cell nuclei and basal membranes in the Scop group (Figure 3(c)). In the Con and EA groups, only a few lipid droplets were in the LG acinar cell. These results indicated that EA *treatment* resulted in a notable reduction in lipid accumulation.

### 4.4. EA Stimulation Regulates Proinflammatory Factors and Chemokines

**Figure 4:**
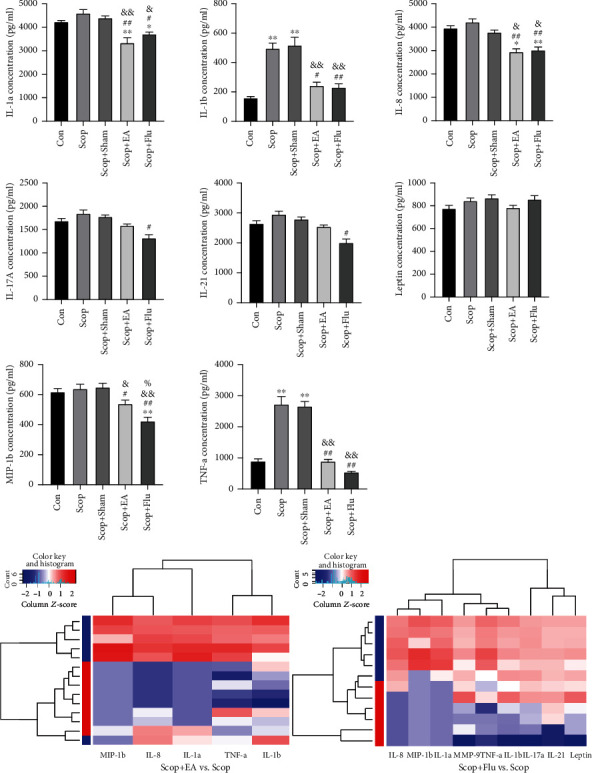
Changes in the cytokines and the chemokines in rabbit LGs on day 35. Quantitative data are expressed as mean ± SEM (*n* = 4). EA stimulus significantly decreased the levels of IL-1a, IL-1b, MIP-1b, TNF-*α*, and IL-8 when compared with the Scop group. The Scop+Flu group significantly decreased the levels of IL-1a, IL-1b, IL-17A, IL-21, MIP-1b, TNF-*α*, and IL-8 when compared with the Scop group. ^∗^*P* < 0.05 and ^∗∗^*P* < 0.01 vs. the Con group; ^#^*P* < 0.05 and ^##^*P* < 0.01 vs. the Scop group; ^&^*P* < 0.05 and ^&&^*P* < 0.01 vs. the Scop+Sham group; ^%^*P* < 0.05 vs. the Scop+EA group. Clustering heat map: blue represents the Scop group.

The protein chip technology was used to evaluate the IL-1a, IL-1b, IL-8, IL-17A, IL-21, Leptin, MIP-1b, and TNF-*α* expression levels in the LG tissue to study the inhibitory effect of the EA stimulation on the inflammatory response induced by proinflammatory factors and chemokines. Compared with those in the Con group, the expression levels of IL-1b and TNF-*α* in the Scop and Scop+Sham groups were significantly upregulated (*P* < 0.01, Figure 4). Compared with those in the Scop group, the IL-1a (*P* < 0.01), IL-1b (*P* < 0.05), MIP-1b (*P* < 0.05), TNF-*α* (*P* < 0.01), and IL-8 (*P* < 0.01) levels dropped significantly after the EA stimulation (Figure 4). Compared with those in the Scop group, the levels of IL-1a (*P* < 0.05), IL-1b (*P* < 0.01), IL-17A (*P* < 0.05), IL-21 (*P* < 0.05), MIP-1b (*P* < 0.01), TNF-*α* (*P* < 0.01), and IL-8 (*P* < 0.01) in the Scop+Flu group decreased significantly (Figure 4). These results showed that EA had an anti-inflammatory effect similar to Flu.

### 4.5. EA Inhibitory Effect on NF-*κ*B Is Dependent on *α*7nAChR

**Figure 5:**
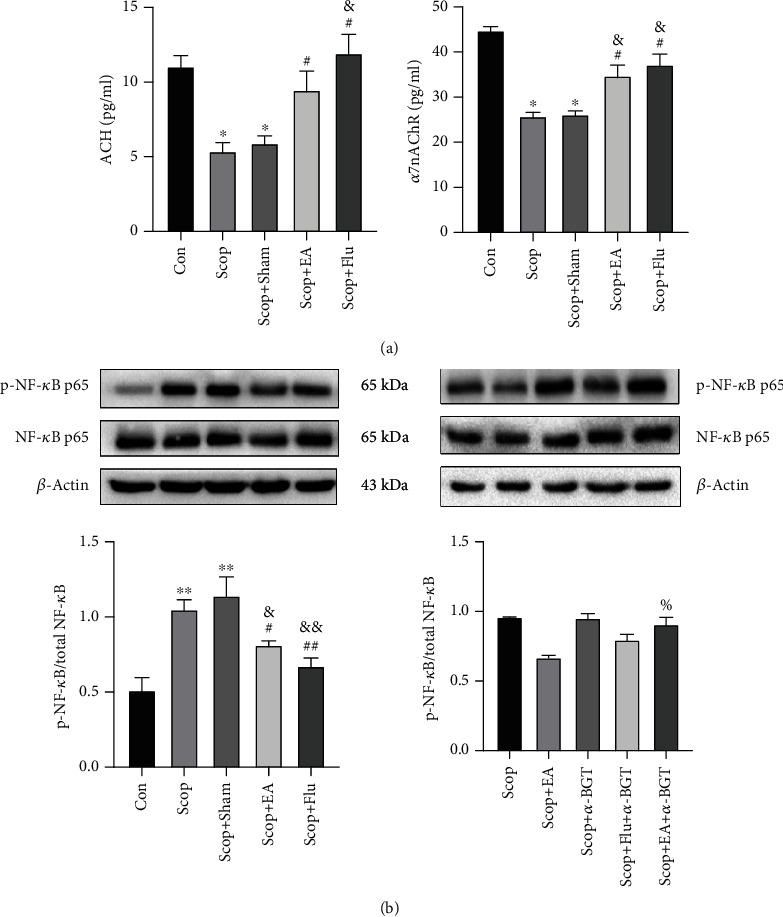
Effect of electroacupuncture on the changes in ACH and *α*7nAChR in the LG determined using ELISA. (a) Western blot of the effect of EA on the activation of NF-*κ*B with *β*-actin as a load control. (b) Quantitative data are expressed as mean ± SEM (*n* = 3). ^∗^*P* < 0.05 and ^∗∗^*P* < 0.01 vs. the Con group; ^#^*P* < 0.05 and ^##^*P* < 0.01 vs. the Scop group; ^&^*P* < 0.05 and ^&&^*P* < 0.01 vs. the Scop+Sham group; ^%^*P* < 0.05 vs. the Scop+EA group.

The *α*7nAChR antagonist *α*-BGT was subjected to ELISA to detect the contents of ACh and *α*7nAChR and Western blot to detect the expression levels of NF-*κ*B p65 and p-NF-*κ*B p65 to verify whether EA regulated *α*7nAChR and participated in the inhibitory effect of NF-*κ*B. Compared with the Con group, the Scop group significantly reduced the contents of ACh (*P* < 0.01) and *α*7nAChR (*P* < 0.01, Figure 5(a)) but increased the expression level of p-NF-*κ*B p65 (*P* < 0.05, Figure 5(b)). Compared with the Scop group, the EA stimulation upregulated the expression levels of ACh (*P* < 0.05) and *α*7nAChR (*P* < 0.05, Figure 5(a)). Compared with that in the EA group, the expression of p-NF-*κ*B p65 in the Scop+EA+*α*-BGT group was upregulated (*P* < 0.05, Figure 5(b)). Therefore, EA could increase the expression levels of ACh and *α*7nAChR in the lacrimal tissue of dry eyes. The deficiency of *α*7nAChR induced by *α*-BGT reversed the inhibition of the NF-*κ*B phosphorylation by the EA stimulus. Flu, a corticosteroid, mimicked the effect of the EA stimulus and inhibited the NF-*κ*B inflammation. These results suggested that the inhibition of NF-*κ*B by the EA stimulus was dependent on *α*7nAChR.

### 4.6. EA Stimulation Regulates Proinflammatory Factors and Chemokines through *α*7nAChR

**Figure 6:**
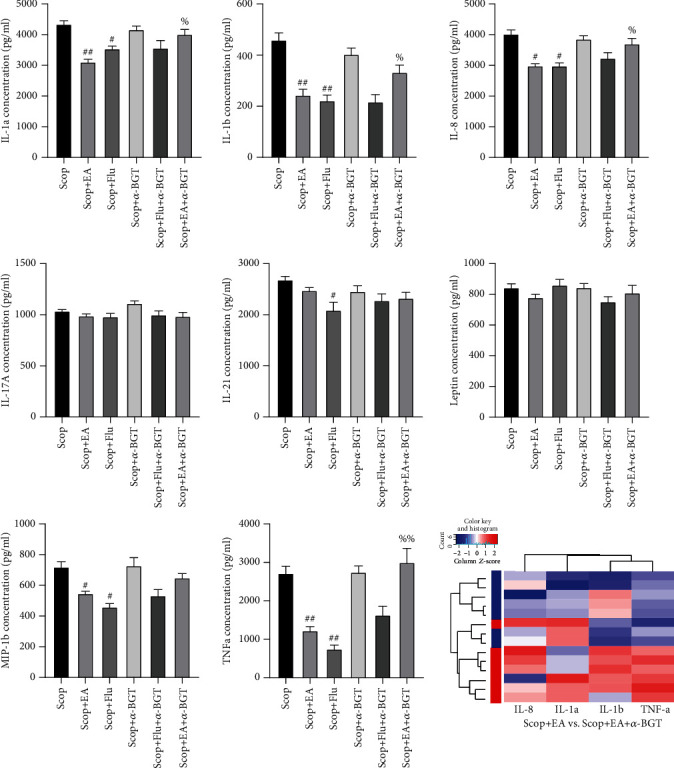
Dependence of the regulation of the electroacupuncture stimulation on inflammatory factors and chemokines on *α*7nAChR. The *α*7nAChR antagonist reverses the inhibitory effect of the EA stimulation on proinflammatory cytokines and chemokines. Quantitative data are expressed as mean ± SEM (*n* = 4). ^#^*P* < 0.05 and ^##^*P* < 0.01 vs. the Scop group; ^%^*P* < 0.05 and ^%%^*P* < 0.01 vs. the Scop+EA group. Clustering heat map: blue represents the EA group.

After using the *α*7nAChR antagonist *α*-BGT to observe whether EA regulated proinflammatory factors and chemokines by using *α*7nAChR, the protein chip technology was used to continue the evaluation of the expression levels of IL-1a, IL-1b, IL-8, IL-17A, IL-21, Leptin, MIP-1b, and TNF-*α* in the lacrimal tissue. Results showed that compared with the EA group, the Scop+EA+*α*-BGT group had upregulated IL-1a (*P* < 0.05), IL-1b (*P* < 0.05), IL-8 (*P* < 0.05), and TNF-*α* (*P* < 0.01) expression levels (Figure 6). Compared with the Scop+Flu group, the Scop+Flu+*α*-BGT group had no significant change in the effects of proinflammatory factors and chemokines (*P* > 0.05, Figure 6). This result showed that the EA stimulation regulated proinflammatory factors and chemokines through *α*7nAChR, whereas the Flu regulation of proinflammatory factors and chemokines did not depend on *α*7nAChR.

### 4.7. *α*7nAChR Is Involved in the Protective Effect of EA on the LG

**Figure 7:**
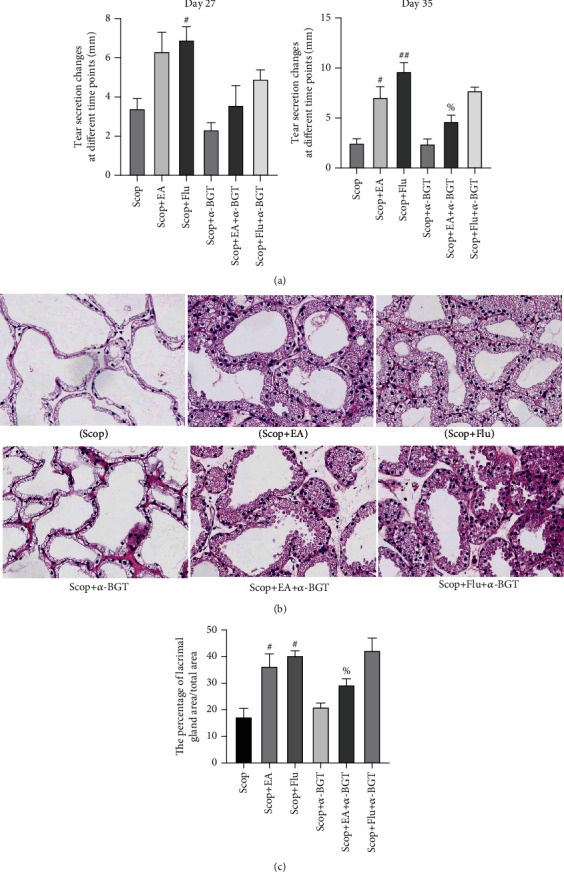
*α*7nAChR is involved in the protective effect of EA on the LG: (a) tear fluid flow (*n* = 6); (b) histopathological images of the cornea (hematoxylin-eosin staining, ×20) on day 35 (*n* = 3); (c) the percentage of lacrimal gland area/total area (%). Quantitative data are expressed as mean ± SEM. ^#^*P* < 0.05 and ^##^*P* < 0.01 vs. the Scop group; ^%^*P* < 0.05 vs. the Scop+EA group.

The tear flow measurement (*n* = 6) and the H&E staining on day 35 were performed to evaluate the area of the LG atrophy (*n* = 3) and detect the involvement of *α*7nAChR on the protective effect of EA on the LG. After 14 days of treatment, compared with that in the Scop group, the tear flow in the EA group increased (*P* < 0.05, Figure 7(a)), which indicated that the function of the LG improved. At the same time, compared with that in the EA group, the tear flow in the Scop+EA+*α*-BGT group was reduced (*P* < 0.05, Figure 7(a)). Compared with the Scop group, the EA group had decreased LG atrophy area after the EA stimulation (*P* < 0.05, Figures 7(b) and 7(c)), and the Scop+Flu group had decreased lymphatic infiltration of the LG. Compared with that in the EA group, the area of the LG atrophy in the Scop+EA+*α*-BGT group significantly increased (*P* < 0.05, Figures 7(b) and 7(c)). These results indicated that EA could reverse the damage of inflammation to the LG. EA and Flu had similar protective effects on the LG, and *α*7nAChR participated in the protective effect of EA on the LG.

## 5. Discussion

In this study, the EA stimulation has shown a protective effect on the corneal epithelium and the LGs of New Zealand rabbits with hydrobromic acid-induced DEDs and can reduce the level of inflammatory cytokines in LGs. The corticosteroid Flu is a commonly used clinical anti-inflammatory drug for the treatment of DEDs. This study has found that the protective and the anti-inflammatory effects of EA on the ocular surface are similar to those of Flu. These beneficial effects are related to the upregulation of *α*7nAChR and the downregulation of NF-*κ*B in the LG. EA and Flu can upregulate the expression of *α*7nAChR and downregulate the expression of NF-*κ*B. The *α*7nAChR antagonist *α*-BGT can reverse the inhibitory effect of EA on the NF-*κ*B pathway and the expression of inflammatory factors but cannot affect the expression of Flu on the NF-*κ*B pathway and inflammatory factors.

Many mechanisms, such as increased tear osmotic pressure, ocular surface inflammatory response and damage [1, 27, 28], oxidative stress [29], and neurological abnormalities [30], induce DEDs. Recently, many studies have mentioned that the inflammation is the main mechanism of the pathogenesis of DEDs [31, 32]. The excessive exposure of the ocular surface to a high-permeability environment and stressful stimuli leads to the excessive production of proinflammatory cytokines and chemokines [33, 34] and further causes the expansion of autoreactive T helper cells and infiltrating the ocular surface and LGs [35, 36], leading to a cycle of ocular surface damage and inflammation. The cholinergic can inhibit inflammation. The cholinergic receptor on lacrimal cells is m3AChR [37]. ACh is the first choice to be combined with m3AChR [38]. Studies have found that [39, 40] after using cholinergic agonists, activating m3AChR promotes the lacrimal protein secretion. However, another study has found that [41] *α*7nAChR relieves the overproduction of LG oxidants caused by radiotherapy and inhibits the inflammation by inhibiting the p38/JNK signaling pathway to protect the tear gland and increase the tissue repair. *α*7nAChR has a therapeutic effect. The therapeutic potential of DEDs in the radiotherapy induced LG damage. The findings of this study have found that *α*7nAChR is also closely related to the pathogenesis of DEDs. As a receptor on macrophages, *α*7nAChR can participate in the protection of LG cells during inflammation and increase the tear secretion.

Inflammation stimulates the activation of the CAP, which is defined as the efferent arm of the vagus nerve of the inflammatory reflex [9]. The efferent activity of the vagus nerve is activated by the release of ACh from the organs of the reticuloendothelial system, and ACh is directly released from the efferent terminal of the vagus nerve in other organs [42, 43]. The cholinergic binds to *α*7nAChR expressed on the surface of activated macrophages and inhibits the NF-*κ*B nuclear translocation through the *α*7nAChR-mediated intracellular signaling pathways to inhibit the production of proinflammatory cytokines [5, 44, 45], ultimately preventing tissue damage. EA can mediate *α*7nAChR to produce anti-inflammatory effects. Wang et al. [46] used EA and acute lung injury rat models to determine whether the former can improve lung injury induced by cardiopulmonary bypass (CPB). Their results show that the stimulating effect of EA can protect against CPB-induced acute lung injury and inhibit the release of HMGB1 through *α*7nAChR activation. Furthermore, it was found that EA stimulation can significantly reduce the area of cerebral infarction and improve neurological deficits, activating *α*7nAChR to reduce the expression of HMGB1, iNOS, IL-1*β*, CD86, TNF-*α*, and IL-6 and increase expression of Arg-1, TGF-*β*1, CD206, IL-4, and IL-10 [47, 48]. Jiang et al. [12] found that EA stimulation attenuated the inflammatory response mediated by the NLRP3 inflammasome after cerebral ischemia/reperfusion (I/R), and the use of *α*7nAChR agonists could induce neuroprotection effects similar to EA stimulation. The above research findings suggest that the anti-inflammatory effect of *α*7nAChR mediated by EA has therapeutic potential for disease intervention. Moreover, EA can also inhibit the expression of the NF-*κ*B signaling pathway and inhibit inflammation through the “cholinergic anti-inflammatory pathway.” According to studies [49], the EA stimulation can upregulate the cylindrical hyperplasia to reduce the inflammatory damage after cerebral ischemia/reperfusion (I/R) and inhibit the NF-*κ*B signaling pathway. A previous study has also found [50] that the EA stimulation of Zusanli (ST36) and the use of *α*7nAChR agonists can alleviate the intestinal I/R injury and reduce NF-*κ*B p65 transcription levels and serum IL-6 and TNF-*α* levels. These observations suggest that EA stimulates the Zusanli to protect the intestinal I/R injury by activating the cholinergic anti-inflammatory pathway. This result is consistent with the results observed in this experiment. EA can relieve DEDs by stimulating acupoints around the eyes. The protective effect of EA on the LG is mediated through the anti-inflammatory pathway, which is mediated by *α*7nAChR. EA can reduce the NF-*κ*B P65 nucleus by regulating *α*7nAChR. The transcription and the reduction in the expression levels of IL-1, MIP-1b, TNF-*α*, and IL-8 and the systemic use of *α*7nAChR antagonists significantly eliminate the anti-inflammatory effects of EA.

This study has used the clinically commonly used anti-inflammatory corticosteroid Flu as a positive control. The potential limitation is the lack of the use of the *α*7nAChR agonist group as a positive control. In future studies, the effects of EA and *α*7nAChR agonists should be considered.

## Data Availability

The data used to support the findings of this study are available from the corresponding author upon request.
